# Activated Müller Cells Involved in ATP-Induced Upregulation of P2X_7_ Receptor Expression and Retinal Ganglion Cell Death

**DOI:** 10.1155/2016/9020715

**Published:** 2016-09-21

**Authors:** Ying Xue, Yuting Xie, Bo Xue, Nan Hu, Guowei Zhang, Huaijin Guan, Min Ji

**Affiliations:** Department of Ophthalmology, Affiliated Hospital of Nantong University, Nantong 226001, China

## Abstract

P2X_7_ receptor (P2X_7_R), an ATP-gated ion channel, plays an important role in glaucomatous retinal ganglion cell (RGC) apoptotic death, in which activated retinal Müller glial cells may be involved by releasing ATP. In the present study, we investigated whether and how activated Müller cells may induce changes in P2X_7_R expression in RGCs by using immunohistochemistry and Western blot techniques. Intravitreal injection of DHPG, a group I metabotropic glutamate receptor (mGluR I) agonist, induced upregulation of GFAP expression, suggestive of Müller cell activation (gliosis), as we previously reported. Accompanying Müller cell activation, P2X_7_R protein expression was upregulated, especially in the cells of ganglion cell layer (GCL), which was reversed by coinjection of brilliant blue G (BBG), a P2X_7_R blocker. In addition, intravitreal injection of ATP also induced upregulation of P2X_7_R protein expression. Similar results were observed in cultured retinal neurons by ATP treatment. Moreover, both DHPG and ATP intravitreal injection induced a reduction in the number of fluorogold retrogradely labeled RGCs, and the DHPG effect was partially rescued by coinjection of BBG. All these results suggest that activated Müller cells may release ATP and, in turn, induce upregulation of P2X_7_R expression in the cells of GCL, thus contributing to RGC death.

## 1. Introduction

Retinal Müller cell activation (gliosis) is a common response in a variety of retinal diseases and injuries, as evidenced by increased expression of glial cytoskeletal proteins, such as glial fibrillary acidic protein (GFAP) and vimentin [1–3]. Previous studies have demonstrated that overactivation of group I metabotropic glutamate receptor (mGluR I) by excessive extracellular glutamate in a rat chronic ocular hypertension model could induce Müller cell gliosis by inhibiting inward rectifying K^+^ channels (Kir) [3–6]. DHPG, a selective mGluR I agonist, may mimic the elevated intraocular pressure-induced changes in GFAP expression in Müller cells [3, 6]. Activated Müller cells may contribute to retinal neurodegeneration through releasing harmful factors, such as tumor necrosis factor (TNF), interleukin-1 (IL-1), nitric oxide (NO), and ATP [1, 7–12].

ATP, released predominantly from glial cells, plays important roles in modulating a variety of physiological and pathological processes by activating Gq-coupled purinergic receptors [13–20]. P2X_7_ receptor (P2X_7_R), one of purinergic (P2) receptors, is an ATP-sensitive ligand-gated cation channel. Activation of P2X_7_Rs leads to ion channel open, which is permeable for small cations (Na^+^, Ca^2+^, and K^+^). Repeated or prolonged activation of P2X_7_Rs under some pathological conditions may result in the formation of a nonselective channel/pore, through which large molecules up to 600–800 Da can pass, thus ultimately leading to cell death [21–23]. It was reported that P2X_7_Rs were expressed in retinal ganglion cells (RGCs), and lots of evidence has demonstrated that activation of P2X_7_Rs may contribute to glaucomatous RGC death [24–26]. In addition, activation of P2X_7_Rs in cultured rat brain astrocytes enhanced metabotropic purinergic receptor P2Y_2_ mRNA expression by a mechanism involving both calcium influx and PKC/MAPK signaling pathways [27]. In the present study, we aimed to explore whether and how activated Müller cells may modulate P2X_7_R expression in RGCs through ATP release.

## 2. Materials and Methods

### 2.1. Animals

All experimental procedures described here were in accordance with the National Institutes of Health (NIH) guidelines for the Care and Use of Laboratory Animals and the guidelines of Nantong University on the ethical use of animals. For intravitreal injection experiments, Sprague-Dawley rats (100–300 g) were obtained from Nantong Laboratory Animal Company and maintained under conditions of a 12 h light/dark cycle. Postnatal 1 d Sprague-Dawley rats were used for primary retinal neuronal cell culture. During this study, all possible efforts were made to minimize the number of animals used and their suffering.

### 2.2. Intravitreal Injection

Rats were anesthetized with an intramuscular injection of a mixture of ketamine (25 mg/kg) and xylazine (10 mg/kg). The pupil was dilated with tropicamide drops, and 10 *μ*M (S)-3,5-dihydroxyphenylglycine (DHPG), 10 *μ*M ATP, or 10 *μ*M BBG dispersed in 2 *μ*L of 0.9% saline was injected into the vitreous space through a postlimbus spot using Hamilton microinjector (Hamilton) under a stereoscopic microscope. The needle was inserted 2 mm behind the temporal limbus and directed to the optic nerve. Eyes that received only an injection of saline in the same manner served as a control group. The eyes with cataract, endophthalmia, or other damage after injection were excluded.

### 2.3. Primary Retinal Neuronal Cell Culture

Primary retinal neuronal cell culture was prepared as described previously [28] with minor modification. Briefly, retinas of newborn Sprague-Dawley rats (1 d) were digested with trypsin (0.25% for 15 min at 37°C) and retinal neurons were dissociated mechanically by using a fire-polished Pasteur pipette. The cell suspension was plated onto 35 mm poly-D-lysine-coated dishes at a density of 1.2 × 10^6^ and cultured in a neurobasal medium (Gibco BRL, Life Technologies, Rockville, MD, USA), supplemented with 2% B27 and 2 mM glutamine, in a humidified 5% CO_2_ incubator at 37°C. RGCs were identified by using antibody against Brn3a, a RGC marker. Experiments were performed on the 8th day of neurons in culture.

### 2.4. Retrograde Labeling and Counting of RGCs

To assess the effect of DHPG on RGCs in vivo, retrograde labeling of RGCs was performed. After anesthetization, the rats were placed in the stereotactic apparatus (Stoelting, Wood Dale, IL, USA) and the brain surface was exposed by perforating the parietal bone to facilitate dye injection. 2 *μ*L of 2% fluorogold (FG; Biotium, Hayward, CA, USA) was injected into both superior colliculi and dorsal lateral geniculate nuclei. After seven days, rats from different groups were sacrificed and eyeballs were enucleated and placed in 4% paraformaldehyde for 4 h. The whole retina was then carefully dissected, flattened, and mounted with the vitreous side up on slides. Photographs were captured using a fluorescent microscope (Leica, Germany) and FG-labeled RGCs were counted in a masked fashion by the same investigator using automated particle counting software in ImagePro Version 6.0 (Media Cybernetics, Bethesda, USA). The number of labeled cells in 12 photographs of each retina (three photographs per retinal quadrant) at 1/6, 3/6, and 5/6 of the retinal radius was summed together and expressed as mean RGC densities/mm^2^ for each group.

### 2.5. Western Blot

Western blot analysis was conducted as previously described with some modifications [3]. After washed with PBS solution, rat retinas or cells were lysed in lysis buffer (containing 1 M Tris-HCl at pH 7.5, 1% Triton X-100, 1% Nonidet p-40, 10% SDS, 0.5% sodium deoxycholate, 0.5 M EDTA, 10 *μ*g/mL leupeptin, 10 *μ*g/mL aprotinin, and 1 mM phenylmethylsulfonyl fluoride). Protein concentrations were determined by the BCA method (Pierce). Protein samples (1.0 g/L, 15 L) were subjected to 10% SDS-PAGE using a Mini-Protean 3 electrophoresis system (Bio-Rad) and electrotransferred to polyvinylidene fluoride membranes using a Mini TransBlot electrophoretic transfer system (Bio-Rad). The membranes were blocked with 5% skimmed milk at room temperature for 1 h and then incubated with rabbit antibody against-P2X_7_R (1 : 1000, Abcam). The blots were washed with TBST and incubated with HRP-conjugated goat anti-mouse or anti-rabbit IgG (1 : 4000, Jackson ImmunoResearch Laboratories) for 1 h at room temperature and visualized with enhanced chemifluorescent reagent ECL (Thermo Scientific, Rockford, IL, USA) and exposed to X-ray film in the dark. The experiments were performed in triplicate, and the protein bands were quantitatively analyzed with Image J Analysis software.

### 2.6. Immunofluorescence Staining

Immunohistochemistry was performed following the procedure described in detail previously [3]. After rats were anesthetized and perfused with 4% paraformaldehyde (in 0.1 M phosphate buffer, pH 7.4), the left eyeballs were removed and postfixed in 4% paraformaldehyde for 2–4 h, followed by dehydration with graded sucrose solutions at 4°C (4 h in 20% and overnight in 30%). The retinas were vertically sectioned at 14 *μ*m thickness on a freezing microtome (Leica, Nussloch, Germany). The slices were collected and mounted on chrome-alum-gelatin-coated slides. After washing with 0.01 M PBS (pH 7.4), the sections were blocked in 6% bovine serum albumin (Sigma, St. Louis, MO, USA) in PBS plus 0.1% Triton X-100 at room temperature for 2 h and then incubated with a rabbit antibody against-P2X7 receptor antibody (1 : 400) or mouse monoclonal against-GFAP (1 : 500, Sigma-Aldrich) antibody at 4°C for 48 h. Immunoreactive proteins were visualized by incubating with FITC-conjugated goat anti-mouse IgG (1 : 200 dilution; Jackson, Immunoresearch Laboratories, Wes Grove, PA, USA). The samples were mounted with anti-fade mounting medium with DAPI (Vector Laboratories, Burlingame, CA, USA) and the immunofluorescence images were visualized with a Leica confocal laser scanning microscope (Leica, Germany).

For immunocytochemistry, cells were washed in PBS and fixed with 4% fresh PFA solution for 40 min at room temperature. Cells were rinsed in PBS, permeabilized with 0.1% Triton X-100, and incubated in PBS containing 1% BSA for 2 h to block the nonspecific binding sites. Triplicate wells were incubated with monoclonal antibodies against Brn3a (1 : 200, Sigma-Aldrich), or a rabbit antibody against-P2X_7_ receptor antibody (1 : 400) at 4°C overnight. On the following day, the appropriate second antibodies and 4,6-diaminodiphenyl-2-phenylindole (DAPI; Sigma- Aldrich) were added in a dark room and incubated for 2-3 h. After washing, preparations were mounted and detected by a fluorescent microscope (Leica, Germany).

### 2.7. Statistical Analysis

All data were expressed as means ± SE. Differences among groups were analyzed by one-way ANOVA, followed by multiple comparison tests (LSD). All statistical analyses were carried out by the aid of SPSS 17.0 software package and significance level was set at *P* < 0.05.

## 3. Results

**Figure 1:**
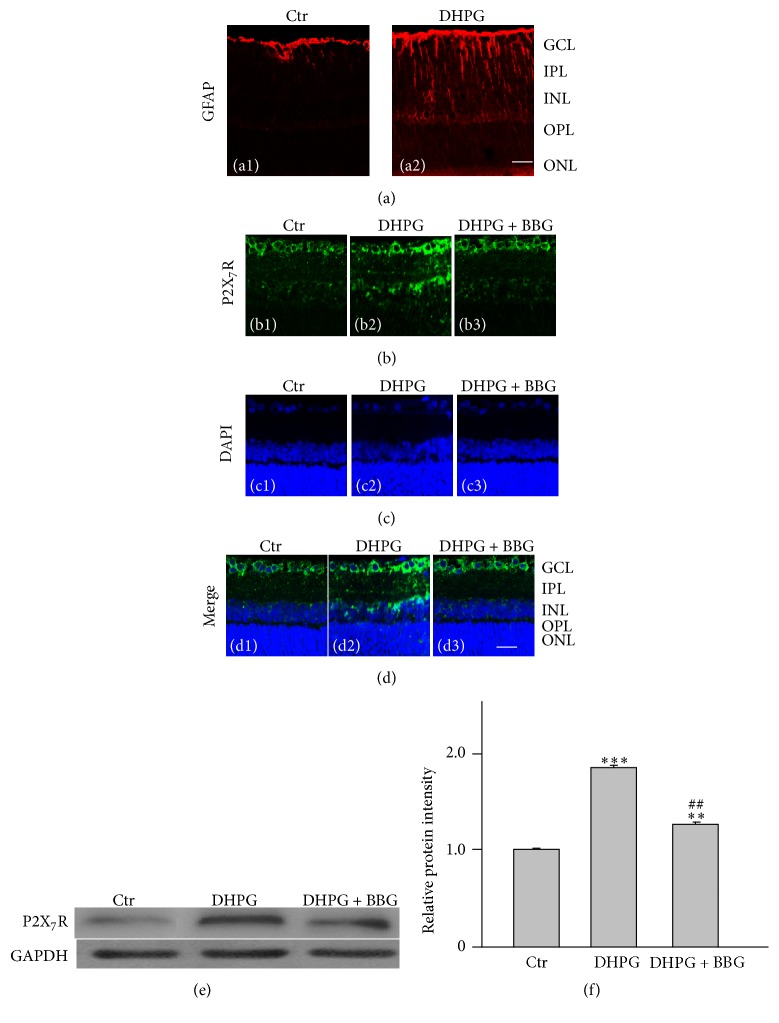
DHPG-induced upregulation of P2X_7_R expression in rat retinas. (a) Immunofluorescence labeling showing the change in GFAP protein expression in rat retinal vertical slices taken from normal physiological solution- (NS-) injected retina (Control, Ctr) (a1) and DHPG-injected retina at 2 weeks after the injection (a2). (b) Immunofluorescence labeling showing the changes in P2X_7_R protein expression in rat retinal vertical slices taken from NS-injected retina (Ctr) (b1), DHPG-injected retina (b2), and BBG + DHPG-injected retina (b3), respectively. (c) (c1)–(c3) are corresponding DAPI images. (d) The merged images of (b1) and (c1) (d1), (b2) and (c2) (d2), and (b3) and (c3) (d3), respectively. Scale bar, 20 *μ*m for all images. GCL, ganglion cell layer; IPL, inner plexiform layer; INL, inner nuclear layer; OPL, outer plexiform layer; ONL, outer nuclear layer. (e) Representative immunoblots showing the changes of P2X_7_R protein expression in control (Ctr), DHPG-injected and DHPG + BBG-injected rats. (f) Bar charts summarizing the average densitometric quantification of immunoreactive bands of P2X_7_R protein levels under different conditions. *n* = 9 for each group. ^*∗∗*^ *P* < 0.01 and ^*∗∗∗*^ *P* < 0.001 versus Ctr; ^##^ *P* < 0.01 versus DHPG alone group.

Our previous study has demonstrated that DHPG, an mGluR I agonist, may induce Müller cell gliosis by inhibiting inward rectifying K^+^ channels [3]. We examined whether Müller cell gliosis by activation of mGluR I may induce changes in P2X_7_R expression in RGCs by using immunohistochemistry and Western blotting. Firstly, we confirmed DHPG-induced Müller cell gliosis as our previous report [3].  Figure 1(a) shows that intravitreal injection of DHPG (10 *μ*M, 2 *μ*L) indeed induced an increase in GFAP expression in the retinal section obtained from 2 weeks after the injection (a2), as compared with that in the normal physiological solution- (NS-) injected retinal section (a1). We then examined changes in P2X_7_R protein expression after the DHPG injection. As shown in Figures 1(b)–1(d), in the NS-injected retinal section (Control, Ctr), P2X_7_R proteins were expressed in cells of the ganglion cell layer (GCL) and the inner nuclear layer (INL), as well as in the outer plexiform layer (OPL) ((b1) and (d1)). P2X_7_R expression showed a remarkable increase in retinal section obtained from the rat at 2 weeks after the DHPG injection, especially in the GCL (Figures 1(b)–1(d), (b2), and (d2)). Since mGluR I expresses extensively in retinal cells, the DHPG-induced upregulation of P2X_7_R expression in the cells of GCL may be mediated by bioactive substances released from activated Müller cells and/or by direct action of DHPG on the cells of GCL. ATP, a P2X_7_R ligand, is one of extracellular signaling molecules for glia-neuron crosstalk, which may be abundantly released from activated Müller cells. We examined whether ATP is involved in the DHPG effect on P2X_7_R expression by acting on P2X_7_Rs. Brilliant blue G (BBG, 10 *μ*M, 2 *μ*L), a specific P2X_7_R antagonist, was intravitreally coinjected with DHPG (10 *μ*M). Retinal slice was made 2 weeks after the injection for immunohistochemistry. As shown in Figures 1(b)–1(d), the DHPG-induced increase in P2X_7_R expression in the cells of GCL in retinal section was significantly reduced by BBG injection ((b3) and (d3)). Similar observations were obtained in other five eyes. Western blotting revealed that total P2X_7_R protein in the DHPG-injected retinas was profoundly increased to 185.0 ± 6.0% of the control, and the protein level was reduced to 121.0 ± 8.0% of the control by coinjection of BBG (*n* = 6, *P* < 0.001) (Figures 1(e) and 1(f)). These results suggest that activated Müller cells induced by DHPG may release ATP, in turn acting on the cells of GCL to upregulate P2X_7_R expression.

**Figure 2:**
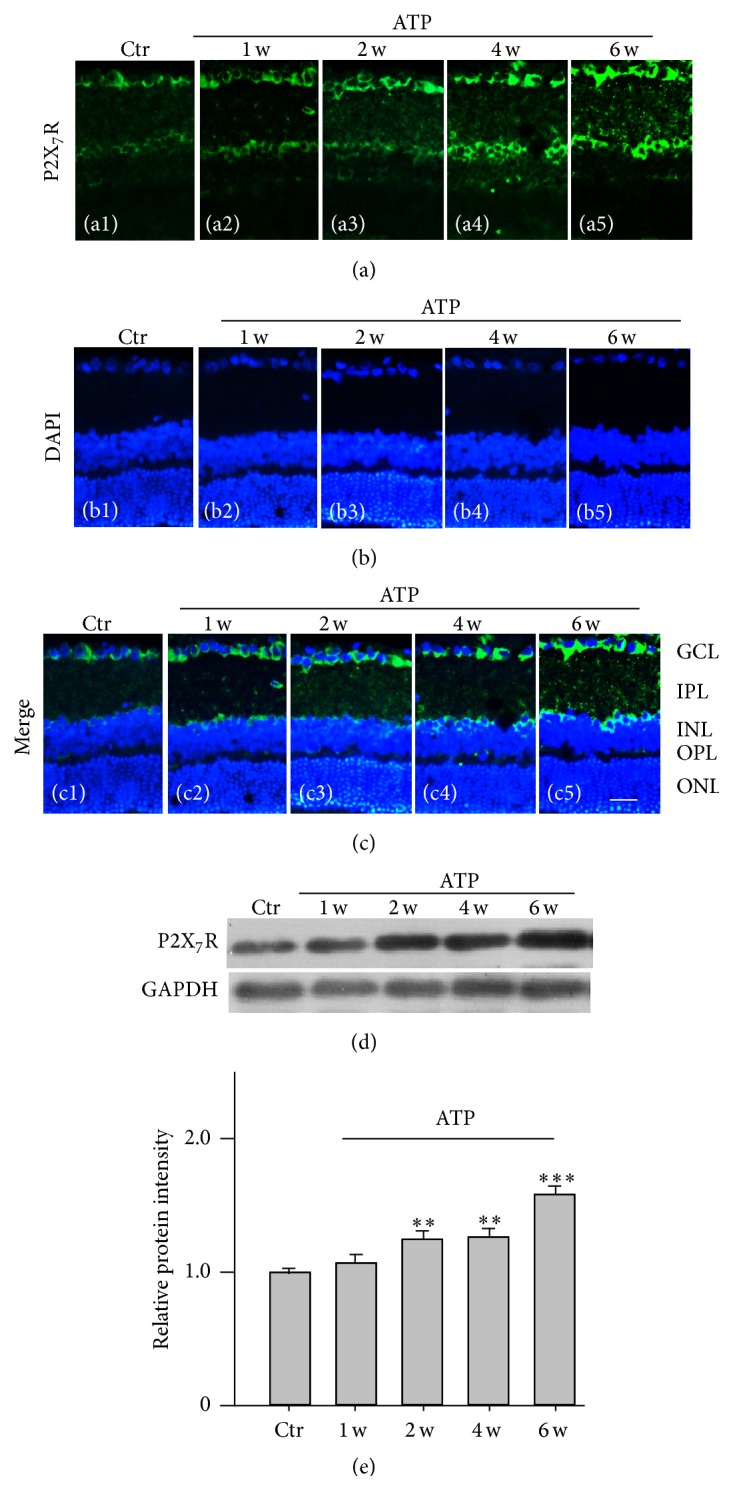
ATP-induced changes in P2X_7_R protein expression in rat retinas. ATP (10 *μ*M, 2 *μ*L) was intravitreally injected every 7 days, and retinal sections or whole retinal extracts were made at different times after the injection. (a) Immunofluorescence labeling showing the changes in P2X_7_R protein expression in rat retinal vertical slices taken from NS-injected retina (Ctr) (a1) and ATP-injected retinas from 1 week (1 w) to 6 weeks (6 w) after the injection ((a2)–(a5)). Note that P2X_7_R protein expression was mainly in the GCL and the INL. (b) (b1)–(b5) show corresponding DAPI images. (c) The merged images of (a1) and (b1) (c1), (a2) and (b2) (c2), (a3) and (b3) (d3), (a4) and (b4) (c4), and (a5) and (b5) (c5), respectively. Scale bar, 20 *μ*m for all images. GCL, ganglion cell layer; IPL, inner plexiform layer; INL, inner nuclear layer; OPL, outer plexiform layer; ONL, outer nuclear layer. (d) Representative immunoblots showing the changes in P2X_7_R protein expression in NS-injected retinas and ATP-injected retinas at different times after the injection. (e) Bar charts summarizing the average densitometric quantification of immunoreactive bands of P2X_7_R proteins under different conditions. *n* = 9 for each group. ^*∗∗*^ *P* < 0.01 and ^*∗∗∗*^ *P* < 0.001 versus Ctr.

We then tested whether ATP treatment may induce changes in P2X_7_R expression. ATP (10 *μ*M, 2 *μ*L) was intravitreally injected every 7 days, and retinal sections or whole retinal extracts were made at different times after the injection for immunohistochemistry and Western blotting, respectively. As shown in Figure 2(a), weak positive signals of P2X_7_R proteins were seen in the retinal section obtained from the NS-injected rat (control) ((a1) and (c1)), especially in the GCL and the INL. P2X_7_R expression was significantly increased in the sections obtained from ATP-injected rats started from 1 week ((a2) and (c2)) and through 6 weeks after the injection ((a3)–(a5) and (c3)–(c5)), as compared with the control (a1). Consistently, Western blot experiments showed that P2X_7_R protein levels were significantly increased after ATP treatment (Figure 2(d)). The average density of P2X_7_R proteins slightly increased (104.1 ± 18.0% of control, *n* = 9, *P* = 0.71) at 1 week after the ATP-injection, while it considerably increased at 2 weeks after the injection (122.7 ± 13.3% of the control, *n* = 9, *P* < 0.01) and through 6 weeks (128.2 ± 8.1% of the control, *n* = 9, *P* < 0.01 at 4 weeks; 156.8 ± 8.8% of the control, *n* = 9, *P* < 0.001 at 6 weeks) (Figure 2(e)). These results suggest that ATP indeed induces upregulation of P2X_7_R protein expression in the cells of GCL.

**Figure 3:**
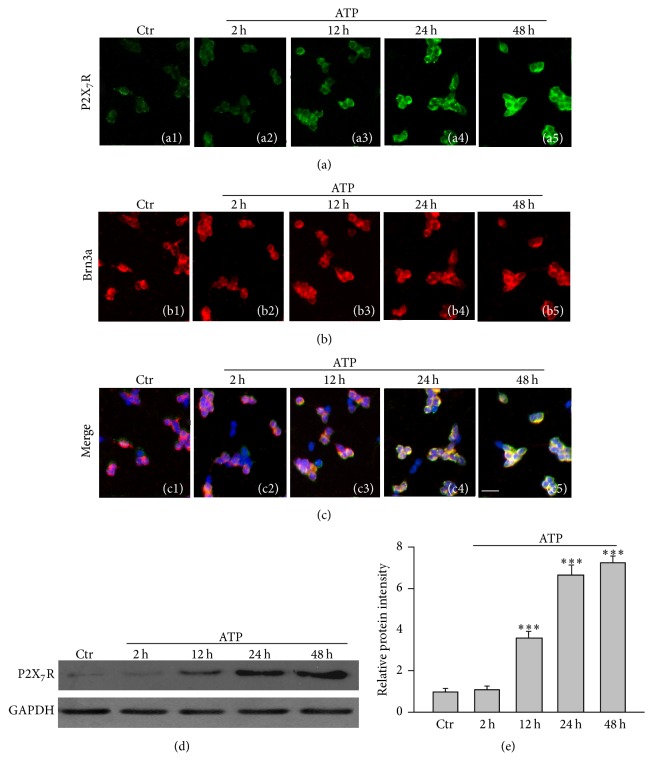
ATP-induced changes in P2X_7_R protein expression in cultured rat RGCs. (a) Immunofluorescence labeling showing the changes in P2X_7_R protein expression in control (Ctr) (a1) and ATP-treated cells for different times (2–48 h) ((a2)–(a5)). (b) Immunofluorescence labeling showing the expression of Brn3a, a RGC marker. (c) The corresponding merged images. Scale bar, 20 *μ*m for all images. (d) Representative immunoblots showing the changes of P2X_7_R protein expression in control (Ctr) and ATP-treated cells for different times (2–48 h). (e) Bar charts summarizing the average densitometric quantification of immunoreactive bands of P2X_7_R protein levels under different conditions. *n* = 9 for each group. ^*∗∗∗*^ *P* < 0.001 versus Ctr.

We further confirmed the ATP-induced upregulation of P2X_7_R expression in primary cultured RGCs. The culture was treated with ATP (100 *μ*M) for different times (2, 12, 24, and 48 h), and P2X_7_R expression was examined by immunocytochemistry and Western blotting.  Figure 3(a) shows representative immunocytochemical results, revealing that P2X_7_R expression did not show a significant change at 2 h after ATP treatment (a1); however, it remarkably increased from 12 h to 48 h after ATP treatment ((a2)–(a5)).  Figure 3(b) shows immunocytochemical staining for Brn3a, a RGC marker. The merged images clearly show that P2X_7_Rs were mainly expressed in Brn3a-positive RGCs (Figure 3(c), (c1)–(c5)). Consistent with these results, Western blot experiments showed that the average density of P2X_7_R proteins was not changed at 2 h after ATP treatment (103.0 ± 12.0% of the control, *n* = 9, *P* = 0.51), but the P2X_7_R protein level considerably increased to 373 ± 23.7% (*n* = 9, *P* < 0.01), 669.3 ± 12.2% (*n* = 9, *P* < 0.001) and 710.2 ± 17.1% of the control (*n* = 9, *P* < 0.001) at 12 h, 24 h, and 48 h after ATP treatment, respectively (Figures 3(d) and 3(e)).

**Figure 4:**
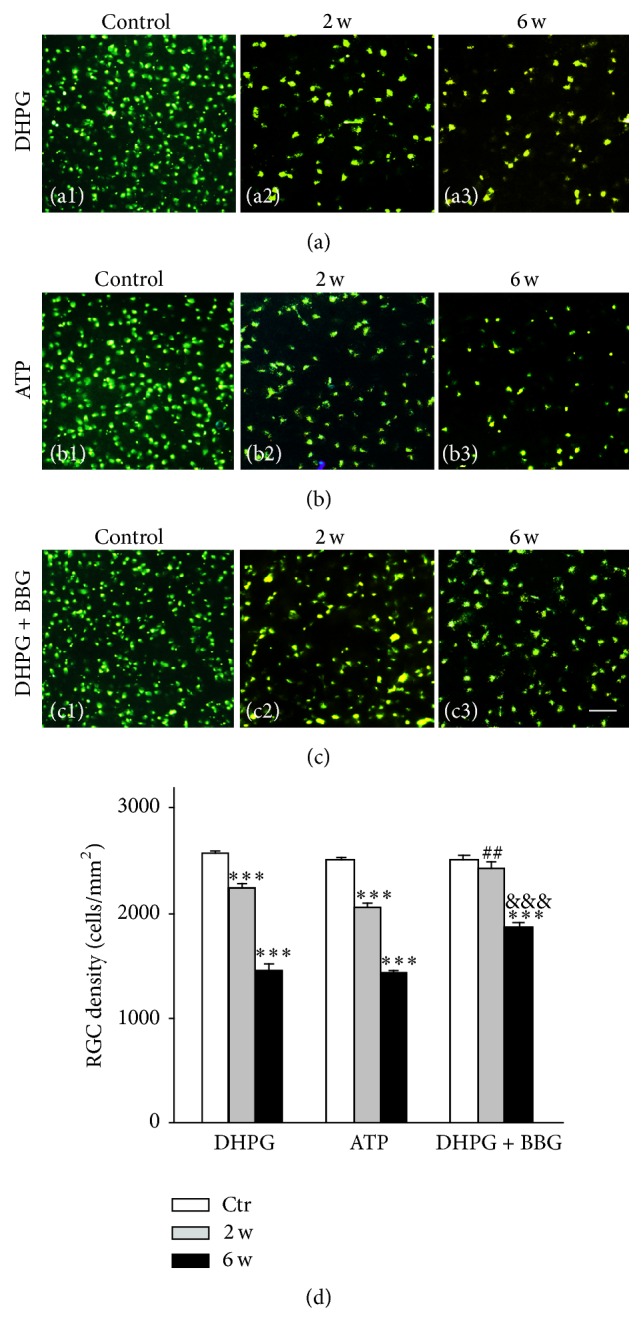
ATP/P2X_7_R-mediated changes in the number of RGCs in DHPG-injected retinas. (a) Confocal laser microphotographs showing the changes in the number of fluorogold (FG) retrogradely labeled RGCs in NS-injected retina (Control, Ctr) (a1), and DHPG-injected retinas at 2 weeks (a2) and 6 weeks (a3) after the DHPG injection, respectively. (b) Confocal laser microphotographs showing the changes in the number of FG-labeled RGCs in Ctr (b1) and ATP-injected retinas at 2 weeks (b2) and 6 weeks (b3) after the ATP injection, respectively. (c) Confocal laser microphotographs showing the changes in the number of FG-labeled RGCs in Ctr (c1), and DHPG + BBG-injected retinas at 2 weeks (b2) and 6 weeks (b3) after the injection, respectively. Scale bar: 100 *μ*m for all images. (d) Bar chart showing the average cell density (cells/mm^2^) under different conditions as shown in ((a)–(c)). *n* = 6 for each group. ^*∗∗∗*^ *P* < 0.001 versus Ctr. ^##^ *P* < 0.01 versus DHPG-treated group at 2 w. ^&&&^ *P* < 0.001 versus DHPG-treated group at 6 w.

Finally, whether ATP may serve as a mediator for Müller cell gliosis and RGC apoptosis was explored by examining changes in number of the FG retrogradely labeled RGCs. To do so, DHPG (10 *μ*M, 2 *μ*L), ATP (10 *μ*M, 2 *μ*L), or DHPG + BBG (10 *μ*M, 2 *μ*L) was intravitreally injected every 7 days, respectively, and the numbers of FG-labeled RGCs were counted at 2 weeks and 6 weeks after the injection. As shown in Figure 4(a), representative micrographs obtained from the flat-mounted retinas at the same angle (0°) [29] illustrated that numbers of FG-labeled RGCs were significantly reduced at 2 and 6 weeks after the DHPG injection ((a2) and (a3)), as compared with the NS-injected retina (a1). Similarly, intravitreal injection of ATP also remarkably reduced the number of FG-positive RGCs (Figure 4(b), (b1)–(b3)). However, the reduction in FG-positive cell numbers in the DHPG-treated retinas was partially rescued by coinjection of BBG (Figure 4(c), (c1)–(c3)). We counted the total number of FG-labeled RGCs in 12 photographs (as mentioned in the methods) and found that the average number of FG-labeled RGCs was 2535 ± 83/mm^2^ in control (*n* = 6), while it declined to 2110 ± 90/mm^2^ (*n* = 6, *P* < 0.001 versus control) and 2037 ± 104/mm^2^ (*n* = 6, *P* < 0.001 versus control) in the DHPG- and ATP-injected retinas at 2 weeks after the injection, and further to 1438 ± 90/mm^2^ (*n* = 6, *P* < 0.001 versus control) and 1367 ± 64/mm^2^ (*n* = 6, *P* < 0.001 versus control) in the DHPG- and ATP-injected retinas at 6 weeks, respectively (Figure 4(d)). Furthermore, the average number of FG-labeled RGCs was rescued to 2329 ± 46/mm^2^ (*n* = 6, *P* < 0.01 versus DHPG alone and *P* = 0.82 versus control) at 2 weeks, and 1799 ± 148/mm^2^ (*n* = 6, *P* < 0.001 versus DHPG alone and *P* < 0.001 versus control) at 6 weeks after coinjection of BBG with DPHG (Figure 4(d)).

## 4. Discussion

In the present study, we found that retinal Müller cell activation induced by the mGluR I agonist DHPG upregulated P2X_7_R expression in the cells of GCL, which was mediated by ATP released from Müller cells, thus contributing to RGC death.

Our previous study has demonstrated that activation of mGluR I induced Müller cell gliosis by inhibiting Kir K^+^ currents, especially Kir4.1 mediated currents, in a rat chronic ocular hypertension model [3]. The mGluR I agonist DHPG treatment of cultured Müller cells or intravitreal injection modulated Kir4.1 proteins and Kir4.1 mRNAs, leading to increase of GFAP expression [3, 6], suggesting that DHPG-induced GFAP expression may be used as a Müller cell gliosis model. In the present study, we confirmed that DHPG injection indeed induced upregulation of GFAP expression in Müller cells. One of our major findings is that DHPG injection significantly upregulated P2X_7_R expression in the cells of GCL in addition to Müller cell activation. The DHPG-induced effect on P2X_7_R expression in the cells of GCL was mediated by ATP released by activated Müller cells. Even though previous study reported that activation of P2X_7_Rs in cultured astrocytes enhanced P2Y_2_ mRNA expression [27], for our knowledge, the present study is the first report, showing the relationship between the activated Müller cells and P2X_7_R expression in RGCs, in which ATP plays a vital role. This is supported by the experimental evidence. Firstly, DHPG injection-induced upregulation of P2X_7_R expression in the cells of GCL was reversed by the P2X_7_R blocker BBG (Figure 1). Secondly, intravitreal injection of ATP may directly induce upregulation of P2X_7_R expression in the cells of GCL (Figure 2). Thirdly, ATP treatment of cultured retinal neurons resulted in upregulation of P2X_7_R expression in RGCs (Figure 3). It should be noted that RGCs also expressed mGluR I; therefore, we cannot exclude a possibility that DHPG may directly activate mGluR I in RGCs and then increase P2X_7_R expression in these cells. However, considering the facts that the DHPG-induced upregulation of P2X_7_R expression was reversed by coinjection of the P2X_7_R blocker BBG (Figure 1) and DHPG-induced reduction in the number of RGCs was partially rescued by BBG (Figure 4), we speculate that the DHPG effects on P2X_7_R expression were mainly mediated by ATP that released from activated Müller cells. Indeed, we have observed that concentrations of extracellular ATP were increased in cultured Müller cells exposed to DHPG (unpublished data). Regarding the mechanisms underlying ATP-induced changes in P2X_7_R expression, one possibility is that under pathological conditions prolonged stimulation of P2X_7_Rs by ATP may increase intracellular Ca^2+^ concentration and then trigger intracellular Ca^2+^-dependent signaling pathways, thus increasing P2X_7_R expression [27, 30].

It is noteworthy that in the present study we did not identify the cell types in the GCL. There was evidence showing that P2X_7_Rs were also expressed in amacrine cells and glial cells [31–36]. Therefore, the increase in P2X_7_R protein expression in the retinal sections, as shown in Figures 1 and 2, may be in displaced amacrine cells, and/or in glial cells in the GCL. However, it should be noted that ATP-induced increase in P2X_7_R proteins was mainly in the GCL and inner nuclear layer (INL) (Figure 2), and ATP could induce an increase in P2X_7_R protein expression in cultured rat RGCs (Figure 3). We speculated that the increased expression of P2X_7_R proteins in the GCL indeed occurred in RGCs, even though we cannot exclude a possibility that these events maybe also occurred in displayed amacrine cells and glial cells.

RGC apoptotic death is a common feature in retinal neurodegenerative diseases, such as glaucoma. It is noteworthy that overactivation of P2X7Rs induced upregulation of P2X7R expression in the cells of GCL (Figure 2), in addition to directly leading to RGC apoptosis. In turn, upregulation of P2X_7_R expression aggravated RGC apoptosis. Therefore, the present study provides evidence that under pathological conditions ATP, released by activated Müller cells, may induce RGC apoptotic death through two pathways, directly acting on P2X_7_Rs and upregulating P2X_7_R expression.

Another important finding in the present study is that both DHPG and ATP intravitreal injection resulted in a reduction in the number of FG-positive RGCs, and the DHPG effect was partially rescued by coinjecting the P2X_7_R blocker BBG. These results provide direct evidence that activated Müller cells in some retinal diseases and injuries contribute to RGC death via elevated ATP/P2X_7_R activity [37–39]. On the other hand, our present results indicate that blocking Müller cell gliosis and inhibiting ATP/P2X_7_R activity may be potentially useful for the therapeutic management of RGC death in retinal diseases and injuries, such as glaucoma.
